# Severe Penile Curvature following Otis Urethrotomy

**DOI:** 10.1155/2013/214082

**Published:** 2013-03-25

**Authors:** Ersagun Karaguzel, Metin Gur, Dogan S. Tok, İlke O. Kazaz, Huseyin Eren, Omer Kutlu, Guner K. Ozgur

**Affiliations:** Department of Urology, Karadeniz Technical University, Faculty of Medicine, 61080 Trabzon, Turkey

## Abstract

Urethral stricture is a common urological pathology with a high recurrence rate after treatment. Urethral manipulations are among its main causes. In this paper, urethral stricture developed secondary to urethral catheterization and was treated with cold-knife internal urethrotomy and the Otis urethrotomy procedure. During the follow-up period, severe ventral penile curvature preventing sexual intercourse developed due to fibrosis of the corpus spongiosum and tunica albuginea of the penis. This ventral penile curvature was corrected with a separate operation using a tunica vaginalis flap harvested from the left scrotum.

## 1. Introduction

Urethral stricture results from fibrosis and scarring that develop in the urethral mucosa and surrounding tissue. Its etiology is mainly due to factors such as transurethral surgeries, urethral catheterization, pelvic trauma, and hypospadias surgery [1]. The treatment of urethral stricture using urethrotomy is still a controversial issue in the current urological literature [2]. Otis urethrotomy has been described as one of the internal urethrotomy techniques [3, 4]. Other therapeutic modalities include urethral dilatation, scar excision, and end-to-end anastomosis, depending on the location and degree of the urethral stricture.

## 2. Case Report

**Figure 1:**
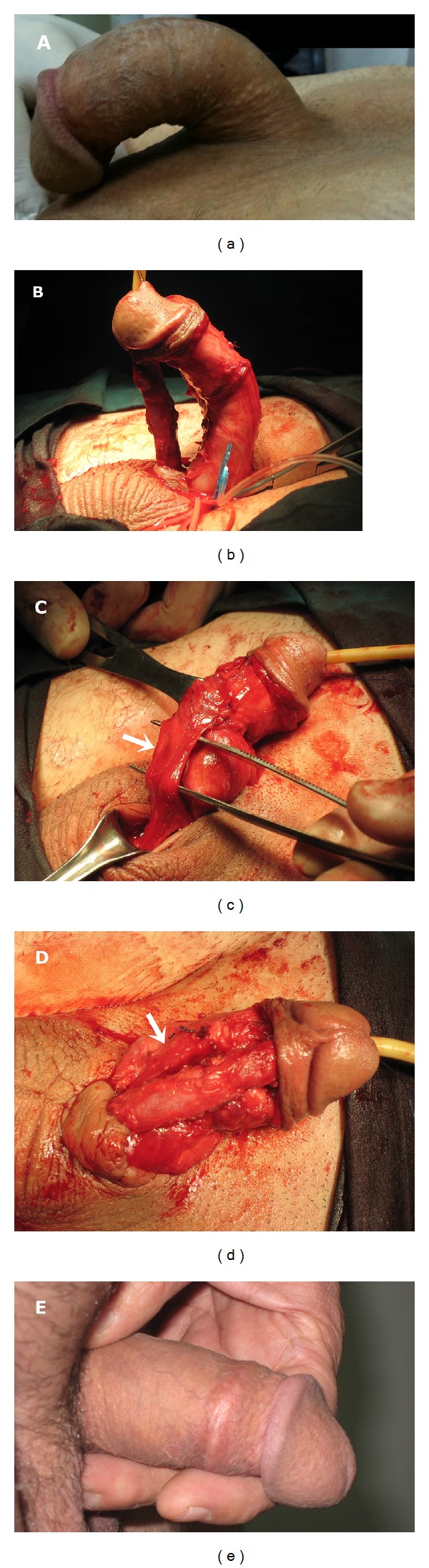
(a) Preoperative appearance of the penile curvature, (b) intraoperative appearance of the penile curvature, (c) importation of the flap of tunica vaginalis, (white arrow), (d) closure of curvature area, which was excised by the flap of tunica vaginalis, (white arrow) and (e) appearance of the penis at 3rd week after the operation.

A 58-year-old man, who had undergone a lobectomy due to lung cancer six months previously in a different center, was referred to our clinic with difficulty in urination. He also had a history of urethral catheterization to monitor urine output during the previous surgery. Uroflowmetry was performed, and the results were consistent with urethral stricture. An almost complete 2 cm urethral stricture was detected at the level of the bulbomembranous urethra during urethroscopy under regional anesthesia. The patient underwent cold-knife internal urethrotomy for the urethral stricture and was discharged in a healthy condition in the postoperative period. Approximately 2 months after the cold-knife internal urethrotomy, the patient represented to our clinic with recurrence of the voiding complaints. Urethroscopy revealed recurred stricture of the bulbomembranous urethra, approximately 3 cm in length, with 6 Fr calibration. The patient underwent cold-knife internal urethrotomy, and deep urethrotomy was performed (up to 30 Fr) in the 12 o'clock position using Otis urethrotomy in order to prevent recurrence. Following Otis urethrotomy and 20 Fr Foley catheter insertion, severe urethrorrhagia was observed and controlled with an external penile bandage. The patient was discharged with no problems on the 3rd day postoperatively. Six months after surgery, no voiding problem was detected, but the patient reported new-onset penile curvature preventing sexual intercourse. A thick-fibrotic band was palpated along the corpus spongiosum at the ventral penis, especially in the midshaft location. Artificial erection was performed by intracavernosal papaverine (50 mg) injection (ICP), and a ventral curvature (approximately 50°) was observed (Figure 1(a)).

To correct the penile curvature, the penis was degloved using a circumcision incision. An 18 Fr Foley catheter was inserted and an artificial erection was established using saline. The urethra was subsequently separated from the penile body (Figure 1(b)). A fibrotic plaque, 2 cm long and 3 cm wide, was seen on the tunica albuginea in the midshaft. We decided to perform plaque excision and to close the penile corpora with a tunica vaginalis flap. After the preparation of tunica vaginalis flap from the left testis, the fibrotic plaque was excised, and the corporeal body was patched with the flap, using watertight absorbable sutures (Figures 1(c) and 1(d)). Additionally, we performed dorsal penile plication using nonabsorbable sutures for the residual curvature. At the 3rd week postoperatively, the patient was reevaluated with penile erection induced with the use of oral phosphodiesterase inhibitor (50 mg sildenafil citrate) and visual sexual stimuli. The figure showed that the penile curvature was completely resolved (Figure 1(e)).

## 3. Discussion

Urethral stricture is one of the most commonly encountered pathologies in daily urology practice, especially in male patients. This can be seen at any age and may be manifested with lower urinary tract symptoms or urinary tract infections that severely impair quality of life. There are various modalities for treatment, depending on the localization, length, and etiology of the urethral stricture. One of the most important iatrogenic causes of the urethral stricture is urethral catheterization [5]. Excessive strain exerted on the catheter along the urethra or inflation of the catheter balloon in the urethra by inexperienced health staff can lead to urethral stricture. In this case, urethral stricture was caused by forced urethral catheterization during lobectomy performed approximately six months before admission to our clinic. The patient, who had no voiding problem before this urethral catheterization, gradually began to experience difficulty in urination during the postoperative period. The patient's symptoms gradually worsened and he experienced difficulties in urination, at which he applied to our clinic.

The literature contains few reports of penile curvature developing after prostatectomy, urethral dilatation, and transurethral prostate and bladder tumor resection [6, 7]. Kelami reported that some patients developed ventral deviation of the erect penis after transurethral manipulations using autophotography in which patients photographed themselves [8]. This condition is known as urethral manipulation syndrome (UMS, Kelami Syndrome). UMS is defined as “an acquired, mostly iatrogenic penile deformity caused by fibrotic changes of the corpus spongiosum” [9]. Inflammation may occur in the corpus spongiosum as a result of traumatic catheterization, recurrent urethral dilatations, cystoscopy, or transurethral surgery. It has also been shown that, following traumatic catheterization procedures, inflammation may develop in the urethra, particularly due to the material used in the urethral catheter [10]. Ventral penile curvature occurs due to the development of fibrosis in the corpus spongiosum. On the other hand, not all patients develop a spongiofibrosis that may lead to penile deviation, even after several urethral manipulations, and why this occurs in a very small number of patients is unclear.

In this case as well, although the urethral stricture was resolved after the surgical intervention, ventral penile curvature occurred due to probable fibrotic process, which developed secondary to instrumentation and interventions in the urethra. We believe that penile curvature resulted from fibrosis that developed in the corpus spongiosum but mostly in the tunica albuginea, especially secondary to deep Otis urethrotomy performed to prevent the recurrence of the urethral stricture. During the surgical procedure performed to correct the penile curvature, in addition to the corpus spongiosum, the tunica albuginea also showed signs of fibrosis. Urethrolysis alone was insufficient to correct the penile curvature, and more complex surgery including fibrotic plaque excision, flap, and penile plication was needed. Deep urethrotomy may exacerbate the fibrotic process and a faster development of fibrosis. Urethrorrhagia developing after Otis urethrotomy was considered as evidence of deep urethrotomy. In addition, bleeding into periurethral tissue may contribute to an excessive fibrotic process.

Consequently, the likelihood of serious ventral penile curvature developing after Otis urethrotomy, one of the methods safely used for the treatment of urethral stricture, should always be kept in the mind, especially in the event of excessive postprocedural bleeding. Great care is required in procedures to be performed blind, such as all forms of urethral manipulation and particularly Otis urethrotomy, and potential urethral damage must be prevented. Urethral damage developing as a result of procedures performed for therapeutic procedures may lead to serious penile curvature at later periods. Correct selection and proper planning of method of treatment are very important in terms of avoiding all these potential complications.
